# Acoustic and language analysis of speech for suicidal ideation among US veterans

**DOI:** 10.1186/s13040-021-00245-y

**Published:** 2021-02-02

**Authors:** Anas Belouali, Samir Gupta, Vaibhav Sourirajan, Jiawei Yu, Nathaniel Allen, Adil Alaoui, Mary Ann Dutton, Matthew J. Reinhard

**Affiliations:** 1 Innovation Center for Biomedical Informatics, Georgetown University Medical Center, Washington, DC USA; 2 War Related Illness and Injury Study Center, Veterans Affairs Medical Center, Washington, DC USA; 3 Department of Psychiatry, Georgetown University Medical Center, Washington, DC USA

## Abstract

**Background:**

Screening for suicidal ideation in high-risk groups such as U.S. veterans is crucial for early detection and suicide prevention. Currently, screening is based on clinical interviews or self-report measures. Both approaches rely on subjects to disclose their suicidal thoughts. Innovative approaches are necessary to develop objective and clinically applicable assessments. Speech has been investigated as an objective marker to understand various mental states including suicidal ideation. In this work, we developed a machine learning and natural language processing classifier based on speech markers to screen for suicidal ideation in US veterans.

**Methodology:**

Veterans submitted 588 narrative audio recordings via a mobile app in a real-life setting. In addition, participants completed self-report psychiatric scales and questionnaires. Recordings were analyzed to extract voice characteristics including prosodic, phonation, and glottal. The audios were also transcribed to extract textual features for linguistic analysis. We evaluated the acoustic and linguistic features using both statistical significance and ensemble feature selection. We also examined the performance of different machine learning algorithms on multiple combinations of features to classify suicidal and non-suicidal audios.

**Results:**

A combined set of 15 acoustic and linguistic features of speech were identified by the ensemble feature selection**.** Random Forest classifier, using the selected set of features, correctly identified suicidal ideation in veterans with 86% sensitivity, 70% specificity, and an area under the receiver operating characteristic curve (AUC) of 80%.

**Conclusions:**

Speech analysis of audios collected from veterans in everyday life settings using smartphones offers a promising approach for suicidal ideation detection. A machine learning classifier may eventually help clinicians identify and monitor high-risk veterans.

## Introduction

Suicide prevention remains a challenging clinical issue, especially among Veterans. According to the most recent data from the United States Department of Veterans Affairs (VA), 17 veterans on average die from suicide per day and rates continue to rise [1]. After controlling for factors like age and gender, Veterans faced a 1.5 times greater risk for suicide compared to adult civilians. From 2005 to 2017, the suicide rate in the US civilian population increased 22.4%, while rates among Veterans increased more than 49% [1]. To help address such alarming rates, there is an urgent need to develop objective and clinically applicable assessments for detecting high-risk individuals. Suicidal ideation is a known risk factor for suicide and has been found to be a predictor of immediate or long-term suicide attempts and deaths [2, 3]. Screening high-risk groups such as veterans for suicidal thoughts is crucial for early detection and prevention [4].

To assess suicidality, healthcare providers use one of the several self-report screening tools such as the Suicidal Ideation Questionnaire (SIQ) or clinician-administered scales, such as the Ask Suicide-Screening Questions (ASQ) or the Columbia-Suicide Severity Rating Scale (C-SSRS) [5–7]. These traditional assessment measures have been found to have marginal predictive validity [8, 9]. Another limitation of these assessments is that they require long visits with a clinician in order to establish rapport [10]. They also rely heavily on a subject’s willingness to disclose their suicidal thoughts. Implicit bias may also affect the mental health assessment process and can lead to wrong screening results [11]. Due to these limitations, research into finding objective markers to aid clinical assessment is key in the fight against suicide.

Recent advances in digital technologies and mHealth devices have the potential to provide novel data streams for suicide prevention research [12]. Speech is an information-rich signal and measurable behavior that can be collected outside the clinical setting, which can increase accessibility to care and enable real-time and context-aware monitoring of an individual’s mental state [13, 14]. Multiple studies have used voice characteristics as objective markers to understand and differentiate various mental states and psychiatric disorders [15]. These include investigations of voice in depression that identified many acoustic markers [13, 16, 17]. In another study, researchers were able to classify depressed and healthy speech using deep learning techniques applied to both audio and text features [18]. Research investigating speech and PTSD in US veterans identified 18 acoustic features and built a classifier to differentiate the 54 PTSD veterans from 77 controls with an area under the ROC curve of 0.95 [19]. A study of bipolar disease collected voice data from 28 patients using smartphones and classified affective states (manic vs depression episodes) longitudinally based on voice features with accuracy in the range of 0.61–0.74 [20]. There is a growing body of literature identifying linguistic patterns that express suicidal ideation [21, 22]. Different computational methods have been employed including feature extraction; topic modeling; word embeddings; traditional as well as deep learning methods to explore and classify suicidality in social media posts [21, 23–25]. Elevated use of absolutist words in tweets has been identified as a marker for anxiety, depression, and suicidal ideation [22]. Other work identified notable word clusters used in the Reddit SuicideWatch forum, which related to suicide risk factors including drug abuse (pills, medication, overdose); and depressive symptoms (pain, angry, sad) [26]. These reports and others support the feasibility and validity of detecting different mental disorders from speech using both acoustic and linguistic features.

Research on the spoken language of suicidal patients dates back as early as 1992, describing suicidal voices as sounding hollow, toneless, monotonous, with mechanical and repetitive phrasing, and a loss in intensity over an utterance [13, 27, 28]. It has been suggested that suicidal mental state causes changes to speech production mechanisms which in turn alter the acoustic properties of speech in measurable ways [28]. Comparisons of suicidal and non-suicidal speech in 16 adolescents identified glottal features as showing the strongest differences between the two groups. In particular, suicidal patients had lower Opening Quotient (OQ), and Normalized Amplitude Quotient (NAQ), acoustic measurements associated with more breathy voices [29]. Acoustic features such as fundamental frequency, amplitude modulation, pauses and rhythm-based features were also used to differentiate between suicidal and depressed patients [17, 30]. Emotion recognition from natural phone conversations was used to classify 43 individuals with and without recent suicidal ideation with an AUC of 0.63 [31]. Similar work on phone conversations in 62 military couples predicted suicidal risk using multimodal features relating to behavior, emotion and turn-taking [32]. Recent work employed both linguistic and acoustic features of speech to classify 379 patients in one of three groups (suicidal, mentally ill but not suicidal, or controls) with accuracies in the range of 0.74–0.85 [33, 34]. Although these are promising findings from different studies, they provide limited details on the acoustic and linguistic variables selected in the models.

Our work investigates speech features in 588 narrative audio diaries collected longitudinally from 124 US Veterans in a naturalistic setting using a mobile app that we developed for data collection. We conducted feature engineering on the recordings to extract sets of features and evaluated different classifiers and learning approaches. This study aims to identify and comprehensively characterize acoustic and linguistic features of speech that could classify suicidal ideation in veterans using audios collected in everyday life settings.

## Materials and methods

### Study data and setting

Data for this work was obtained as part of a larger intervention study for Gulf War Illnesses at the Washington DC VA Medical Center. One hundred forty-nine veterans meeting the Center for Disease Control’s criteria for Gulf War Illness [35] were recruited and of these, 124 participants submitted 588 recordings via an Android smartphone app developed for data collection. The remaining 25 participants did not submit any recordings and were excluded from the analysis. An Android tablet (Samsung Galaxy Table 4) with the mobile app installed was provided to each veteran to enable participation from home.

All data was collected longitudinally from veterans in a naturalistic setting using the smartphone app. At each time-point of the study (week 0, week 4, week 8, 3 months, 6 months, 1 year), participants received reminder notifications and were prompted to complete multiple assessments, which included several self-report psychiatric scales and questionnaires. Veterans responded via audio recordings to open-ended questions about their general health in recent weeks/months and about their expectations from the study.

Each recorded response included a Patient Health Questionnaire (PHQ-9) administered as part of the health questionnaire battery. Item-9 of the PHQ-9 [36] is commonly used in research to screen for suicidal ideation and has been validated to be predictive of suicide in both the general population and in US veterans [37, 38]. It asks, “Over the last two weeks, how often have you been bothered by thoughts that you would be better off dead or of hurting yourself in some way?” Response options are “not at all”, “several days”, “more than half the days”, or “nearly every day”. We considered a subject as suicidal at the time of recording if they answered with any option other than “not at all”.

### Feature extraction and preprocessing

**Fig. 1:**
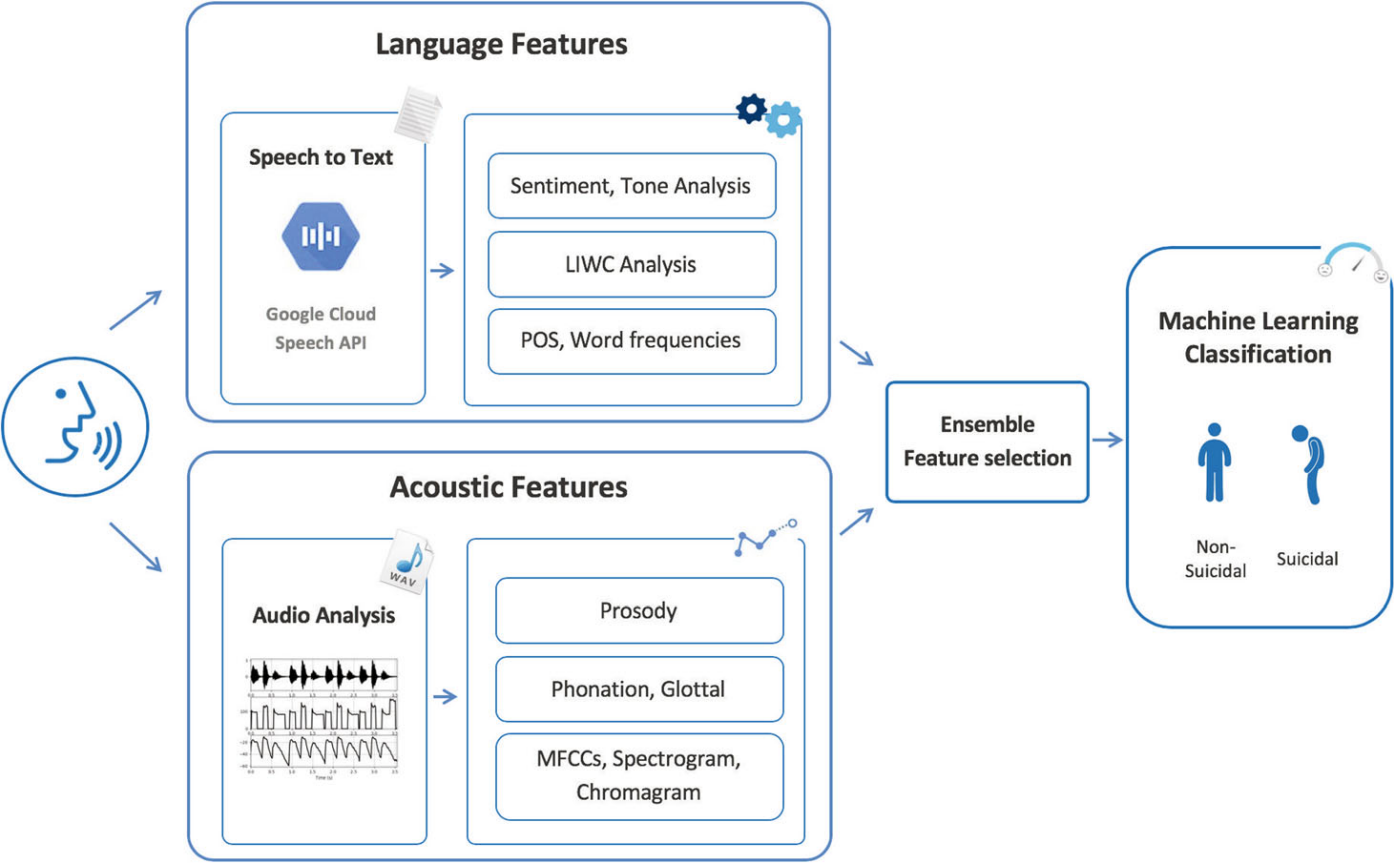
Outline of the study procedure. Acoustic features were extracted using pyAudioAnalysis and DisVoice audio python libraries. Audios were transcribed using Google Speech-to-Text API. Linguistic features were extracted using LIWC. POS and word frequency features were extracted using NLTK. Sentiment and tone analysis was performed using NLTK, Watson Tone Analyzer, Azure Text Analytics, and Google NLP. We perform an ensemble feature selection to identify a subset of predictive features. We use different machine learning and deep learning techniques to build a suicidal ideation classification model

Speech features can be divided into acoustic and linguistic features. We conducted comprehensive feature engineering on each recording to extract several sets of features. The study procedure is outlined in Fig. 1.

#### Acoustic features

A variety of acoustic parameter sets have been proposed for voice research and effective computing [39, 40]. Features including frequency; energy; amplitude; and Mel-Frequency Cepstral Coefficients (MFCC) have been used to classify several mental health states including suicidal ideation [13, 15, 29, 41]. We extracted a total of 508 acoustic features from each recording using two audio signal analysis python libraries: pyAudioAnalysis [42]; and DisVoice [43]. Feature sets from both libraries have been previously used to classify psychiatric disorders and pathological speech [15, 44–46].

We used pyAudioAnalysis [42] to extract short-term feature sequences using a frame size of 50 milliseconds and a frame step of 25 milliseconds (50% overlap). Then, we calculated recording level features as statistics on the short-term features (mean, maximum, minimum, median, standard deviation). The pyAudioAnalysis features include: zero crossing rate, energy and entropy of energy, chroma vector and deviation, spectral features composed of centroid, spread, entropy, flux, rolloff, and MFCCs. Using DisVoice, we computed prosodic features from continuous speech based on duration, fundamental frequency (F0), and energy. Phonation-based features were computed from sustained vowels and continuous speech utterances. For continuous speech, we computed the degree of unvoiced segments in addition to seven descriptors over voiced segments (first and second derivative of F0, jitter, shimmer, amplitude perturbation quotient, pitch perturbation quotient, logarithmic energy), then we derived higher-order statistics for each recording (mean, std., skewness, kurtosis). From sustained vowels, we computed 9 glottal features including: variability of time between consecutive glottal closure instants (GCI); average and variability of opening quotient (OQ) for consecutive glottal cycles; average and variability of normalized amplitude quotient (NAQ) for consecutive glottal cycles; average and variability of H1H2: difference between the first two harmonics of the glottal flow signal; and average and variability of Harmonic richness factor (HRF). In addition, four higher-order statistics were derived (mean, std., skewness, kurtosis).

#### Linguistic features

All audio files were automatically transcribed using the Google speech-to-text API, that achieves above 95% accuracy in speech recognition tasks [47]. We manually verified transcriptions for 10% of the audios and while there were a few errors in the transcriptions, there were no major errors that changed the meaning of the answers. We did not correct the transcribed text corpus manually, as one of our goals was to assess the feasibility of an automated approach of both acoustic and linguistic analysis of speech. Subsequently, we used the transcribed text and various Natural Language Processing (NLP) techniques to extract different sets of textual features.

##### Parts of speech (POS)

We use the NLTK library [48] to compute POS frequencies in the transcribed text. POS counts include word classes and lexical categories. Furthermore, we computed word frequencies of absolutist terms which have been associated with suicidal ideation in previous research [22].

##### Sentiment analysis

Given the psychological nature of suicidal ideation, assessing the general polarity and emotions of the recordings is necessary. We compute sentiment scores and emotion level scores to detect joy, fear, sadness, anger, analytical, confident, and tentative tones in the language used by veterans. Sentiment analysis was performed using the following tools and APIs: NLTK, IBM Watson Tone Analyzer, Azure Text Analytics, and Google NLP. Most sentiment analysis tools are developed using text from reviews and tweets, which are different from transcribed text of audio recordings to open-ended questions about veterans’ general health. Hence, we didn’t limit our feature extraction to a single tool. We aim to obtain a better estimate of the valence and emotions through feature selection and weighting of the combination of sentiment features.

##### Linguistic Inquiry and Word Count program (LIWC)

The LIWC software [49] is a text analysis tool that has been extensively used in the mental health space to explore various text corpora for hidden insights from linguistic patterns. The program produced 94 features per recording, based on validated dictionaries covering a wide range of categories to assess different psychological, affective, and linguistic properties.

##### Text visualization

We used Scattertext [50], a text visualization tool to understand differences in speech between suicidal and non-suicidal veterans. The tool uses a scaled f-score, which takes into account the category-specific precision and term frequency. While a term may appear frequently in both groups, the scaled f-score determines if the term is more characteristic of one category versus another. Stopwords such as “the”, “a”, “an”, “in” were excluded from the corpus.

### Statistical analysis

We computed a total of 679 acoustic and linguistic features to understand speech in veterans with suicidal ideation. To compare suicidal and non-suicidal speech, we investigated these features by checking their statistical significance and magnitude of effect size. We used chi-square test for categorical variables and kruskal-wallis *H-*test for both continuous and ordinal variables. Raw *p*-values (*p*-raw) were adjusted for multiple testing using the Bonferroni correction where *p*-adj = *p-raw* x n, where n is the number of independent tests. We define statistical significance as *p-adj* < 0.05. We calculated the effect size using *epsilon*-squared (ϵ^*2*^) to understand the influence of individual variables [51, 52]. The goal of this first analysis is to infer any significant relationships between the characteristics of speech and suicidal ideation.

### Machine learning model development

We performed a second analysis on the extracted feature set using Machine Learning (ML). ML is an analytical approach that can uncover hidden and complex patterns to help generate actionable predictions in clinical settings [53]. An essential step of any ML procedure is feature selection to reduce redundant variables and identify a stable subset of features. This can help create models that are easier to interpret and implement in real-life settings. We implemented an ensemble feature selection approach to select the top performing features across multiple selectors. This approach is known to improve the robustness of the selection process, especially in cases of high-dimensional and low sample size [54]. In particular, we used algorithms with built-in feature importance or coefficients such as ridge, lasso, random forest, and recursive feature elimination using logistic regression. For each algorithm, the best subset of features is selected and scores are assigned to each single feature. A mean-based aggregation is used to combine the results and calculate a mean-score for every feature. The mean-score provides a ranking of the top important and stable features. We used this score to retain different subsets of features using different thresholds. We evaluated model performance based on features with a ranking above the threshold. The best model performances used the top 15 ranked features. .

We observed class imbalance in our dataset with 1 suicidal recording for every 6 non-suicidal. To computationally deal with this imbalance, we used the SMOTE technique [55] to oversample the minority class in the training sets after partitioning the data during the learning process. It is essential to oversample after data partitioning to keep the test data representative of the original distribution of the dataset and avoid information leakage that can lead to overly optimistic prediction results [56].

We investigated six different supervised classification algorithms on the selected features and evaluated the results. The algorithms were: logistic regression (LR); random forest (RF); support vector machines (SVM); XGBoost (XGB); k-nearest neighbors (KNN); and deep neural network (DNN). Prediction performance was assessed using the area under the receiver operating characteristic curve (AUC), which indicates how capable a model is at distinguishing between classes. Although this metric can be optimistic for imbalanced datasets, it still shows a relative change with better performing models, especially when higher sensitivity is desired (i.e., detection of suicidal recordings is more important) [57]. Additionally, we report sensitivity, specificity, F1 score, positive predictive value (PPV), negative predictive value (NPV), and accuracy. Since our data is imbalanced, it was important to assess the performance of the models based on all the metrics jointly. We used the Youden index [58] to identify the optimized prediction threshold to balance sensitivity and specificity.

For model evaluation and selection, we performed a nested cross-validation (CV) learning approach where we split the data into a 5-fold inner and a 5-fold outer CV sets. During each iteration of the nested CV, we kept 1 outer fold for testing (20% of the samples) and used the 4 remaining folds in the 5-fold inner CV to search for the optimal model. We used a grid-search method in the inner loop to tune the different classification algorithms across a wide range of their respective hyperparameter settings. The final generalization error was estimated by averaging AUC scores over the outer test splits. We used nested CV, as opposed to regular k-fold CV, to reduce overfitting and produce stable and unbiased performance estimates that can generalize to unseen data [59–61].

The data partitioning applied during the nested CV was stratified. This means that each fold of the CV split had the same class distribution as the original dataset (1:6 ratio). Further, given the longitudinal aspect of the dataset, multiple recordings can belong to the same participant and may have different suicidal ideation labels across time. This potentially introduces data leakage where recordings from the same participant end up in both training and test folds. Since our goal was to build participant-independent models, we conducted a subject-wise CV to mirror the clinical use-case scenario of screening in newly recruited subjects [62]. Subject-wise CV ensures that recordings from the same patient will not appear in two different folds.

We built 3 different models to assess the predictive performance of acoustic and linguistic features separately and also when combined. All variables were normalized to a range of zero to one before fitting the models. The recordings were considered independent of the type of question asked or when they were recorded. In addition, we evaluated different minimum word counts and minimum audio length cutoffs for the inclusion of the recordings in the modeling.

## Results

### Demographics and recordings characteristics

Between May 2016 and January 2020, 124 participants submitted 588 recordings via the data collection mobile app. The average age of this group was 52.4 years (std = 9.4) and the majority of participants were male veterans (79%).

Table 1. Presents a breakdown of audio recordings by suicidality at each time-point of the study. Out of 588 audios, 504 were non-suicidal and 84 suicidal. All veterans recorded audios at least once, 27 veterans (21.7%) recorded a maximum of eight recordings, and 74 veterans (59.6%) recorded at least 4 recordings. During week 0 and week 8, participants were asked to record two separate audios. Suicidal audios prevalence by time-point ranged from 10.8% at baseline to 23.6% at 3 months. Out of 124 patients 34 (25.8%) were suicidal at some point of the study. Eight patients were suicidal at every time point when they submitted a recording. Twenty-six patients converted between suicidal and non-suicidal ideation. After transcribing the audios, 15 recordings had no text transcriptions (5 suicidal and 10 non-suicidal). These audios were then manually verified and eventually excluded from the study, as they were either empty or had short intelligible speech. Results of the statistical analysis on the acoustic and linguistic features are summarized in Table 2.

**Table 1:** Breakdown of audio recordings by suicidality at each time-point of the study

Time-point	Non-suicidal *N* = 504	Suicidal *N* = 84	Total audios *N* = 588	Participants *N* = 124
Week 0, n (%)^a^	214 (89.2)	26 (10.8)	240	120
2 weeks follow-up, n (%)	63 (87.5)	9 (12.5)	72	72
8 weeks follow-up, n (%)^a^	100 (84.7)	18 (15.3)	118	50
3 months follow-up, n (%)	42 (76.4)	13 (23.6)	55	55
6 months follow-up, n (%)	45 (86.5)	7 (13.5)	52	52
1 year follow-up, n (%)	40 (78.4)	11 (21.6)	51	51

Audios are labeled using item-9 of the PHQ-9 assessment completed at each time-point

^a^During week 0 and week 8, participants recorded two audios

**Table 2:** Summary statistics of significant features for suicidal and non-suicidal groups

Variables	Overall mean (std)	Non-suicidal mean (std)	Suicidal mean (std)	p-adj	ϵ^**2**^
Time between GCIs - mean	0.0039 (0.0009)	0.0038 (0.0009)	0.0043 (0.0011)	0.03	0.03
Time between GCIs - std	0.0072 (0.0028)	0.0074 (0.0029)	0.0060 (0.0019)	0.05	0.03
Energy contour for voiced segments - std	2.8089 (0.9230)	2.8806 (0.9270)	2.3614 (0.7624)	< 0.001	0.04
Energy contour for voiced segments - skewness	−0.6763 (0.3286)	−0.7059 (0.3288)	− 0.4916 (0.2613)	< 0.001	0.05
Energy contour for voiced segments - kurtosis	0.0866 (0.7950)	0.1583 (0.8101)	−0.3609 (0.5018)	< 0.001	0.06
Energy contour for voiced segments - average tilt of a linear estimation	−34.9081 (24.2386)	−36.2156 (24.4906)	−26.7487 (20.9610)	0.04	0.03
Energy contour for voiced segments - average MSE of a linear estimation	5.7945 (3.4302)	6.0285 (3.4929)	4.3348 (2.5833)	0.01	0.03
Delta energy entropy - std	0.3577 (0.0788)	0.3631 (0.0759)	0.3240 (0.0878)	0.04	0.03
Delta MFCC11 - std	0.1881 (0.0172)	0.1893 (0.0169)	0.1805 (0.0169)	< 0.001	0.04
Delta MFCC12 - std	0.1823 (0.0154)	0.1832 (0.0152)	0.1761 (0.0154)	0.01	0.03
Delta MFCC1 - std	1.0386 (0.2200)	1.0525 (0.2112)	0.9515 (0.2528)	0.05	0.03

Abbreviations: *GCI* Glottal closure instants, *MSE* Mean squared error, *std*. Standard deviation, *MFCC* Mel-Frequency Cepstral Coefficients, *p-adj* Adjusted *p*-value

### Acoustic analysis

Average audio length was 44.19 s (std = 52.27). There were no significant differences between suicidal and non-suicidal recordings in audio length, overall audio loudness, or duration of pauses.

Suicidal recordings were mainly different from non-suicidals in terms of energy. Suicidal speech had a lower standard deviation of energy contours for voiced segments *(p-adj < 0.001,* ϵ^*2*^ *= 0.04)*, a lower kurtosis *(p-adj < 0.001,* ϵ^*2*^ *= 0.06), and a* skewness closer to zero *(p-adj < 0.001,* ϵ^*2*^ *= 0.05)* which reflect respectively flatter, less bursty, and less animated voice.

Suicidal speech had lower voiced tilt *(p-adj = 0.04,* ϵ^*2*^ *= 0.03)* and less energy entropy (*p-adj = 0.04,* ϵ^*2*^ *= 0.03*) thus displaying less vocal energy and less abrupt changes. Suicidal speech also exhibited a lower standard deviation of delta MFCC11 *(p-adj = 0.004,* ϵ^*2*^ *= 0.03),* delta MFCC12 *(p-adj = 0.004,* ϵ^*2*^ *= 0.03),* and delta MFCC1 *(p-adj = 0.05,* ϵ^*2*^ *= 0.03)*. The decrease in time derivatives (delta) of MFCC coefficients indicates a lack of variance of energy over time in suicidal speech which can be interpreted as dull and more monotonous voices. Additionally, suicidal veterans produced speech that was irregular in time and displayed high variability between consecutive GCIs (*p-adj = 0.035,* ϵ^*2*^ *= 0.03)*, which can be interpreted as breathier voices.

### Linguistic analysis

Average word count was 70.96 words per recording (std = 93.76) with an average of 15.05 words per sentence *(std = 9.62)*. There were no significant differences between suicidal and non-suicidal recordings in word count or words per sentence. Suicidal participants used more possessive pronouns *(p-raw = 0.005,* ϵ^*2*^ *= 0.005*) and more superlative adverbs *(p-raw = 0.005,* ϵ^*2*^ *= 0.008)*. The analysis of the LIWC scaled scores showed that suicidal participants also used more family references (e.g., daughter, dad, aunt) *(p-raw = 0.014,* ϵ^*2*^ *= 0.010)* and more family male references (e.g., boy, his, dad) *(p-raw < 0.001,* ϵ^*2*^ *= 0.021).* Non-suicidal recordings contained more agentic language (e.g., win, success, better) *(p-raw = 0.035,* ϵ^*2*^ *= 0.007)*. There were no significant differences between the two groups in sentiment scores or usage of negative or positive emotion words*.* After adjusting for multiple testing, no linguistic features were significant.

**Fig. 2:**
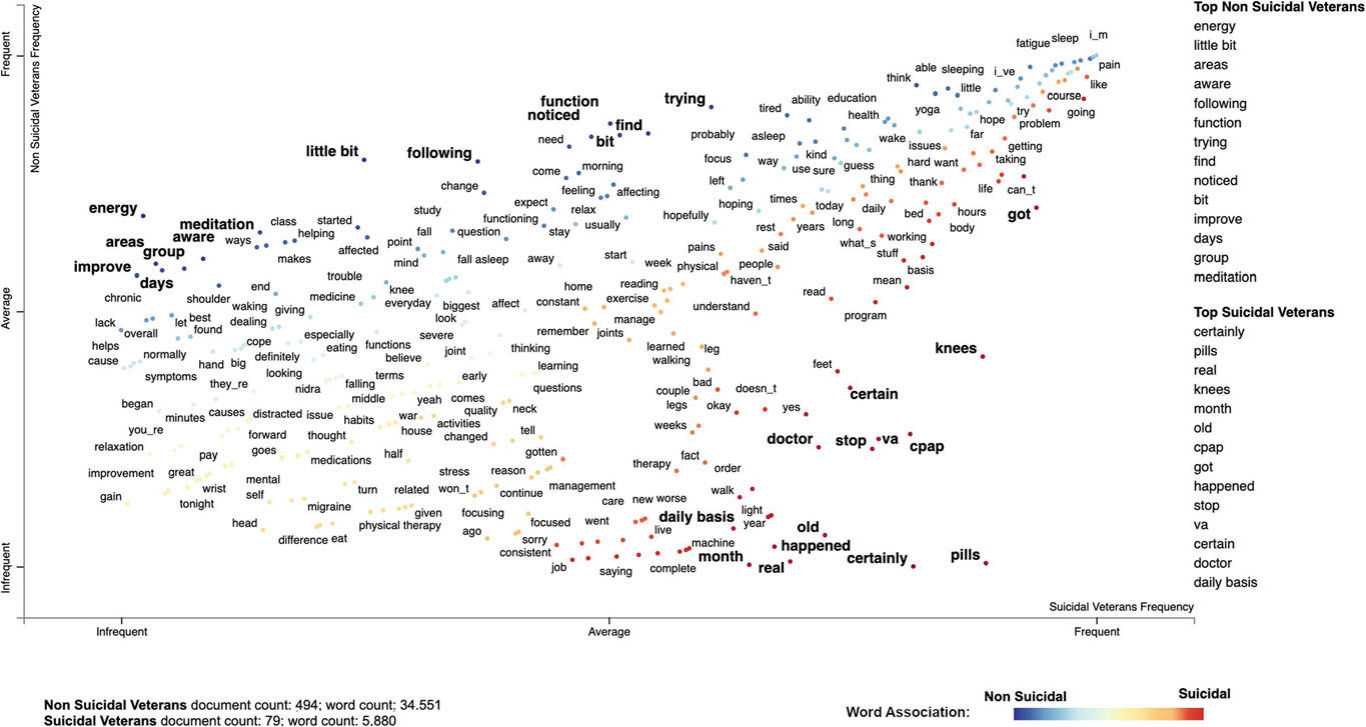
Scattertext visualization of words associated with both suicidal and non-suicidal ideation groups. The red dots on the right lower side of the plot represent terms that are more associated with the suicidal ideation group compared to the blue dots which indicate terms associated with the non-suicidal ideation group

Scattertext analysis (Fig. 2) shows the top words used by both suicidal and non-suicidal veterans. Over 40 thousand words from the text corpus were analyzed to assign a scaled f-score to each word. Ranking words by f-score can help identify which terms are more characteristic of suicidal ideation versus non-suicidal ideation. Top words used by veterans experiencing suicidal thoughts were: “certainly”; “pills”; “real”; “knees”; “month”; “old”; “CPAP; “got”; “happened”; “stop”; “VA”; “certain”; “doctor”; “daily basis”. Top words used by non-suicidal veterans were: “energy”; “little bit”; “areas’; “‘aware”; “following”; “function”; “trying”; “find”; “noticed”; “bit”; “improve”; “days”; “group”; “meditation”.

### Selected features and prediction performance

Table 3 presents 15 acoustic and linguistic features retained by the ensemble feature selection approach. These variables were used for the combined modeling (acoustic + linguistic) which yielded the best results. Out of the selected features, four were linguistic and assessed the use of superlative adverbs, possessive pronouns, proper nouns, and agentic language. The remaining features were acoustic and related to energy dynamics, MFCC, F0, and glottal flow measurements (i.e., OQ, NAQ).

**Table 3:** Description of the top 15 acoustic and linguistic features (rank-ordered by importance) retained for the combined machine learning modeling

Feature	Type	Description
**Delta energy entropy (max)**	Acoustic (Prosody)	Entropy of energy can be interpreted as a measure of abrupt changes.
**Delta energy (mean)**	Acoustic (Prosody)	Dynamic changes in energy relate to the perceptual characteristics of pitch and loudness.
**Energy contour (kurtosis)**	Acoustic (Prosody)	The kurtosis of the energy contour for voiced segments.
**Delta F0 (std)**	Acoustic (Prosody)	First derivative of the fundamental Frequency. Reduced F0 can indicate low pitch and a flatter voice.
**MFCC5 (max)**	Acoustic (MFCC)	5th MFCC coefficient. It can describe vocal tract changes in voice spectral energy.
**Superlative adverbs**	Linguistic (POS)	Use of superlative adverbs (e.g., biggest, hardest, etc.)
**OQ (skewness)**	Acoustic (Glottal)	OQ is a measurement of the glottal flow. It can differentiate between a breathy and tense voice.
**Achievement language**	Linguistic (LIWC)	Use of agentic language as defined by the LIWC achievement dictionary (words such as win, success, better)
**Delta chroma 2 (min)**	Acoustic (Prosody)	A representation of spectral energy. Chroma-based features are also referred to as “pitch class profiles“.
**Proper nouns**	Linguistic (POS)	Use of singular proper nouns.
**Delta energy (median)**	Acoustic (Prosody)	Dynamic changes in energy relate to the perceptual characteristics of pitch and loudness.
**Delta chroma 1 (median)**	Acoustic (Prosody)	A representation of spectral energy. Chroma-based features are also referred to as “pitch class profiles“.
**Delta MFCC5 (max)**	Acoustic (MFCC)	The frame-based delta of the 5th MFCC coefficient. It can measure vocal tract changes.
**NAQ (mean)**	Acoustic (Glottal)	Average NAQ is a measure of the glottal flow that can differentiate between a breathy and tense voice.
**Possessive pronouns**	Linguistic (POS)	Use of possessive pronouns (e.g., my, his, hers)

Abbreviations: *F0* Fundamental frequency, *NAQ* Normalized Amplitude Quotient, *OQ* Opening Quotient, *std*. Standard deviation, *MFCC* Mel-Frequency Cepstral Coefficients, *POS* Parts of speech

We evaluated different word count (WC) cutoffs to identify the minimum utterances needed to better discriminate suicidal ideation in recordings. Classification performances increased as we increased the WC minimum. The best classification results were obtained for recordings with a minimum of 25 words.

Table 4 presents the performance of the classifiers. The XGB classifier on acoustic features reached an overall AUC of 0.77 (std = 0.08) with a sensitivity of 0.67 (std = 0.18), specificity of 0.74 (std = 0.21), F1 equal to 0.43 (std = 0.12), and accuracy of 0.73 (std = 0.17). The LR classifier performed slightly better in terms of AUC with 0.78 (std = 0.12) and sensitivity of 0.78 (std = 0.11) but had a lower F1 equal to 0.38 (std = 0.08), lower specificity of 0.64 (std = 0.15), and a much lower accuracy of 0.66 (std = 0.13).

**Table 4:** Classification results for suicidal ideation based on acoustic and linguistic features

Feature set	Model	Specificity (std)	Sensitivity (std)	PPV (std)	NPV (std)	Accuracy (std)	AUC (std)	F1 (std)
**Acoustic**	**KNN**	0.52 (0.23)	0.78 (0.13)	0.21 (0.03)	0.94 (0.06)	0.55 (0.19)	0.69 (0.11)	0.33 (0.07)
	**LR**	0.64 (0.15)	0.78 (0.11)	0.26 (0.02)	0.95 (0.05)	0.66 (0.13)	0.78 (0.12)	0.38 (0.08)
	**RF**	0.64 (0.12)	0.76 (0.17)	0.25 (0.03)	0.94 (0.05)	0.65 (0.09)	0.76 (0.06)	0.36 (0.04)
	**SVM**	0.54 (0.30)	0.74 (0.18)	0.21 (0.04)	0.93 (0.07)	0.56 (0.25)	0.63 (0.25)	0.33 (0.10)
	**XGB**	0.74 (0.21)	0.67 (0.18)	0.29 (0.04)	0.93 (0.06)	0.73 (0.17)	0.77 (0.08)	0.43 (0.12)
	**DNN**	0.70 (0.06)	0.66 (0.20)	0.26 (0.03)	0.93 (0.03)	0.70 (0.05)	0.75 (0.06)	0.35 (0.08)
**Linguistic**	**KNN**	0.69 (0.15)	0.38 (0.14)	0.16 (0.03)	0.87 (0.05)	0.65 (0.11)	0.52 (0.07)	0.22 (0.03)
	**LR**	0.57 (0.24)	0.66 (0.20)	0.20 (0.05)	0.91 (0.07)	0.58 (0.18)	0.62 (0.06)	0.29 (0.04)
	**RF**	0.64 (0.17)	0.76 (0.09)	0.26 (0.09)	0.94 (0.08)	0.65 (0.14)	0.74 (0.07)	0.38 (0.09)
	**SVM**	0.63 (0.33)	0.48 (0.40)	0.13 (0.09)	0.88 (0.08)	0.61 (0.24)	0.49 (0.15)	0.19 (0.14)
	**XGB**	0.70 (0.16)	0.67 (0.17)	0.27 (0.08)	0.93 (0.05)	0.69 (0.13)	0.72 (0.04)	0.37 (0.06)
	**DNN**	0.50 (0.15)	0.66 (0.25)	0.16 (0.04)	0.90 (0.06)	0.52 (0.11)	0.63 (0.14)	0.25 (0.07)
**Acoustic and Linguistic**	**KNN**	0.61 (0.22)	0.78 (0.22)	0.26 (0.09)	0.94 (0.06)	0.63 (0.19)	0.69 (0.15)	0.37 (0.11)
	**LR**	0.74 (0.14)	0.78 (0.19)	0.35 (0.14)	0.95 (0.05)	0.75 (0.11)	0.77 (0.12)	0.46 (0.13)
	**RF**	0.70 (0.16)	0.84 (0.09)	0.32 (0.08)	0.96 (0.08)	0.72 (0.13)	0.80 (0.06)	0.45 (0.09)
	**SVM**	0.86 (0.13)	0.52 (0.32)	0.32 (0.21)	0.92 (0.05)	0.82 (0.09)	0.64 (0.27)	0.38 (0.24)
	**XGB**	0.79 (0.11)	0.74 (0.18)	0.37 (0.11)	0.95 (0.05)	0.78 (0.08)	0.77 (0.05)	0.47 (0.07)
	**DNN**	0.68 (0.06)	0.70 (0.15)	0.24 (0.04)	0.93 (0.03)	0.68 (0.04)	0.77 (0.08)	0.36 (0.05)

On linguistic features, RF performed better than other classifiers overall with a sensitivity of 0.76 (std = 0.09), specificity of 0.64 (std = 0.17), accuracy of 0.65 (std = 0.14), F1 equal to 0.38 (std = 0.09), and an overall AUC of 0.74 (std = 0.07). XGB classifier performed better in terms of accuracy = 0.69 (std = 0.13) and specificity = 0.70 (std = 0.16) but performed lower on sensitivity = 0.67 (std = 0.17), F1 = 0.37 (std = 0.13), and AUC = 0.72 (std = 0.04).

Combining both acoustic and linguistic features improved most of the models in Table 4. RF classifier correctly identified suicidal ideation in veterans with an overall sensitivity of 0.84 (std = 0.09), specificity of 0.70 (std = 0.16), accuracy of 0.72 (std = 0.13), F1 equal to 0.45 (std = 0.09), and an AUC of 0.80 (std = 0.06). XGB performed better than RF in terms of F1 reaching a score of 0.47 (std = 0.07), specificity of 0.79 (std = 0.11), and accuracy of 0.78 (std = 0.08). XGB performed much lower in sensitivity 0.74 (std = 0.18) compared to RF. Overall, tree-based models (RF and XGB) performed best on this dataset.

## Discussion

In this study, we conducted a two-part analysis. First, we investigated the importance of the extracted acoustic and linguistic features using statistical significance. Second, we evaluated an ensemble feature selection approach to identify a subset of features that can be used in an ML model to detect suicidal ideation in veterans. The results demonstrate that characteristics of speech can be useful in differentiating between suicidal and non-suicidal recordings. Our findings also indicate that audios collected outside the clinical setting, using a mobile app, can be used to classify suicidal ideation with an overall AUC of 0.80, 86% sensitivity, and 70% specificity. This translates to a false positive rate (FPR) of 30% and a false negative rate (FNR) of 14%. At baseline out of 120 patients (107 non-suicidal and 13 suicidal), the model is able to identify 11 suicidal patients out of 13 while misclassifying 32 non-suicidal patients as suicidal. An FPR of 30% remains high but could be improved by targeting only veterans at high risk. Given that audio collection using an app is low cost, easy to administer, and safe, an improved model could be acceptable to both patients and practitioners if appropriate follow-up of individuals with positive screening is ensured. This is essential to avoid causing harm to inadvertently flagged veterans who may not benefit from an intervention.

In the first analysis, we sought to understand characteristics of suicidal speech in veterans and infer significant relationships. We identified 3 higher order statistics (std, kurtosis, skewness) related to energy contour of voiced segments. These variables displayed the largest effect size and indicated the strongest difference between the suicidal and non-suicidal groups. Veterans with suicidal ideation spoke in voices that were flatter, less bursty, and less animated. Additional energy-based variables such as speech tilt, energy entropy, and MFCC coefficients, indicated that speech in suicidal veterans had less vocal energy, less abrupt changes, and was more monotonous. The analysis of the glottal flow parameters related to GCIs indicated a more breathy voice quality in suicidal veterans. These findings are in line with results from previous work on other risk groups. For example, a study that examined GCIs, OQ, and NAQ, found that suicidal adolescents had a more breathy voice quality when compared to non-suicidal adolescents [29]. In addition, multiple research studies used levels of energy dynamics and MFCC features to distinguish controls and depressed subjects who subsequently attempted suicide [17, 28, 63]. A recent study using crowd-sourced audios from the web also identified MFCC features and F0 as important voice biomarkers for suicidal ideation [64]. The general dullness of speech and reduction in energy has also been correlated with PTSD in veterans compared to controls [19].

The linguistic analysis, including sentiment analysis, produced no significant variables. This may be due to the fact that the sentiment tools we used are pre-trained on text corpora such as reviews or tweets, which is different from the transcribed text of the study. Nevertheless, we observed trends indicating more superlative adverbs, possessive pronouns, and proper nouns in suicidal speech. On the other hand, non-suicidal veterans used more agentic language based on the LIWC achievement score. The scatter text analysis, although exploratory in nature, provided frequently used words among non-suicidal and suicidal veterans. Overall, non-suicidal veterans used action verbs and words indicating improvements (e.g., function, improve, trying, find, noticed, aware). Whereas, we found that suicidal veterans spoke with certainty (e.g., certain, certainly), discussing topics such as chronic pain (pills, knees) or sleep problems (CPAP machine), when describing their general health in the past weeks and months. Interestingly, chronic pain and apnea have been linked to suicide as risk factors [65, 66]. Previous research on internet forums showed that suicidal subjects use more possessive pronouns and more absolutist words [22, 67]. It is important to note that the frequently used terms identified in this analysis may come up in general assessment and be benign, and hence are not readily useful for assessment outside of technological approaches.

The second part of our analysis was building a classification model for suicidal ideation. The results show that acoustic-based models performed better (AUC = 0.78) than models based on linguistic features alone (AUC = 0.74). Given the links between suicidal ideation and language, we also explored advanced NLP techniques to improve the linguistic models, such as word and document embeddings. Classification using embeddings provided weak results (not presented), which was mainly due to the relatively small text corpus. Such techniques can be promising for the classification of suicidal ideation if applied to a much larger corpus. We achieved higher AUCs by combining both acoustic and linguistic features (AUC = 0.80). This is in line with previous research on depression and other mental states where fusion of different modalities such as audio, text, and visuals helped improve prediction results [18, 68–70]. AUCs reached by our models are comparable to previous research on suicidal ideation and speech in other risk groups, however, the few published studies either relied on smaller sample size or didn’t discuss important features that went into the final prediction models [17, 29, 33, 71, 72].

Most previous work investigating speech in suicidal ideation uses structured or semi-structured clinical interviews, which may introduce interviewer biases [13]. In this study, we collect free speech recordings longitudinally from US veterans in a real-life setting using a mobile app. Collecting data digitally without the involvement of another person can reduce the stress associated with the fear of being judged and hence produce less biased recordings. Additionally, subjects using a mobile app might be willing to disclose more [73, 74]. Such an approach has the potential to be implemented for longitudinal and context-aware monitoring by collecting audio diaries from veterans at high risk. An application could be developed to collect audio diaries from veterans at high risk and share an assessment of suicidal ideation with a trained healthcare provider as a report. We acknowledge that as a standalone system, models could lead to a high rate of false positives. However, these systems do not aim to provide a diagnosis and are intended to support clinical decision making by augmenting the screening process.

The impact of findings from this study may be limited by a number of factors. We relied on self-report to indicate whether a subject was suicidal or not at the time of the recording. Hence, it is possible that some of the recordings were mislabeled if a participant was not willing to divulge their suicidal ideation. Additionally, the simplification of suicidal ideation to binary labels, suicidal or not, does not consider that ideation might exist on a spectrum. Further, audios ranged from a few seconds to several minutes long and were made in a variety of everyday life settings which could have introduced background noise and quality issues. We believe that a quality check module could be developed within the data collection app and implemented separately during the audio recording step. This can help assess speech audibility and prompt subjects to re-record or move to a place that is quieter. An additional limitation may stem from possible confounders such as demographics (gender, sociolect, etc.) or other mental states that participants might suffer from such as depression; PTSD; or anxiety. This makes it difficult to determine whether the identified features are solely linked to suicidal ideation or might be linked to other comorbid mental states that are more likely to present in suicidal subjects. Future work conditioning on gender and assessing other mental states along with suicidal ideation could help improve the classifiers and further validate the identified features. Improvements to the classifiers could also come from different fusion methods of acoustic and linguistic features such as ensemble modeling or from context-based analysis where the questions asked are also weighted in the models.

## Conclusions

We showed that speech analysis is a promising approach for detecting suicidal ideation in veterans. We also demonstrated that recordings collected longitudinally outside the clinical setting, using a mobile app, can be utilized for such analysis. Using predictive modeling, we identified a set of important acoustic and linguistic markers of speech that can be useful in classifying suicidal ideation in these recordings. The choice of the ML approach and dimensionality reduction techniques were important to optimize the performance of the classifiers and provide realistic estimations on unseen data. Further external validation and optimization are needed to validate and improve these findings. Overall, our work supports the feasibility of an automated approach of both acoustic and linguistic analysis of speech in everyday life settings, which holds the promise for real-time suicidal ideation assessment in high-risk veterans.

## Data Availability

The datasets generated during and/or analyzed during the current study are not publicly available due to content that potentially identifies participants.
